# Evaluating a cost-effective, web-based AI platform for lateral cephalometric analysis: a comparative in-silico study

**DOI:** 10.1186/s12903-025-07108-6

**Published:** 2025-12-18

**Authors:** Omar Ali Kamel Eldeeb, Yomna Mohamed Yacout, Hassan Mohamed Abouelkheir, Yousria Salah ElDin Gaweesh, Noha M. Elkersh

**Affiliations:** 1 Master’s candidate, Department of Oral Medicine, Periodontology, Oral Diagnosis and Oral Radiology, Faculty of dentistry, Alexandria University, Alexandria, Egypt; 2 Department of Orthodontics, Faculty of Dentistry, Alexandria University, Alexandria, Egypt; 3 Department of Oral Medicine, Periodontology, Oral Diagnosis and Oral Radiology, Faculty of Dentistry, Alexandria University, Alexandria, Egypt

**Keywords:** Artificial intelligence, Lateral cephalogram, Steiner analysis, Tweed analysis

## Abstract

**Background:**

Despite the current popularity of artificial intelligence (AI)- assisted cephalometric analysis, its cost precludes its widespread use in low-income countries. Free alternatives are available but mostly lack rigorous validation. Thus, this study aimed to assess the precision of AI-assisted tracing using a freely available web-based platform (WebCeph) (Artificial Intelligence Orthodontic & Orthognathic Cloud Platform, South Korea) by comparing it with the gold standard (manual tracing on OnyxCeph software) (Image Instruments, Chemnitz, Germany). It was also compared with commercially available AI-assisted tracing software (Carestream) (Carestream Dental LLC, Atlanta, GA, USA).

**Materials and methods:**

Lateral cephalometric radiographs of 57 randomly selected patients were included in this study. The radiographs were obtained from the archives of the Radiology unit, Faculty of Dentistry, Alexandria University. Three lateral cephalometric analysis methods were used. OnyxCeph3™ software was considered the gold standard (Control group), the web-based, WebCeph™ platform, was used as an online AI-based cephalometric analysis platform and set as test group 1, and the desktop-based, Carestream imaging software V8, was used for AI cephalometric analysis and set as test group 2. Skeletal and dental measurements of Steiner and Tweed analyses that were generated using the three methods were compared.

**Results:**

Statistically significant differences were detected between the three methods in 11 out of the 18 cephalometric measurements. Notably, in Steiner analysis, the Max1-NA angle was significantly larger in WebCeph than Onyxceph and Carestream by 2.38° and 3.41°, respectively. The Mand1-NB angle was significantly larger in Onyxceph than Carestream and WebCeph by 3.07° and 2.01°, respectively. In Tweed analysis, all the measured variables were significantly different between the three methods, except the AFH. Particularly, the values of IMPA, POr-OcP, PFH, and facial height index were significantly different in Carestream than both Onyxceph and WebCeph. Fewer and smaller discrepancies were detected between WebCeph and the manual gold standard than those detected between Carestream and the manual gold standard.

**Conclusions:**

The freely available AI web-based platform, WebCeph, performed comparably to manual tracing, and outperformed the commercially available AI desktop-based software, Carestream, in several key parameters, making it a viable tool for resource-limited settings.

**Supplementary Information:**

The online version contains supplementary material available at 10.1186/s12903-025-07108-6.

## Introduction

Since its introduction, lateral cephalometric radiography has been used primarily to assess dental and skeletal changes. Currently, it plays a significant role in orthodontic diagnosis and treatment planning [1]. As it enables the evaluation of the skeletal relationships between the maxilla, mandible, and cranial base in both vertical and sagittal planes, along with the dental relationships between the upper and lower teeth and the skeletal structures [2]. Conventional cephalometric analysis involves tracing of anatomical landmarks on an acetate paper followed by measurement of the cephalometric parameters. This method is time-consuming and heavily relies on the experience and knowledge of the clinician [3]. 

Recent software developments and advancements in machine learning have led to a digital revolution and to the introduction of the Artificial intelligence (AI) era. AI is the machine’s capability to imitate human behavior, including complex activities and making decisions based on preset standards [4]. Its use is rapidly evolving in many fields. In the medical field it is mainly used in gastroenterology, cardiology, and radiology [5–7]. In dentistry, AI is used for several functions, such as labeling teeth, spotting cavities, identifying periapical conditions, assessing the relationship between the mandibular canal and third molars, analyzing osteoporotic alterations, planning dental implants, investigating jaw tumors, and carrying out cephalometric analysis [8, 9]. 

Convolutional neural networks (CNNs) are specialized versions of machine learning methods that are the most commonly employed algorithms for image analysis in AI [10, 11]. One of the forms of image analysis using AI is AI-aided cephalometric analysis. The AI-aided cephalometric analysis has gained attention because it minimizes manual tracing-related problems such as subjectivity, limited reproducibility, and being an operator-dependent time-consuming process [12, 13]. Automated landmark localization has been the subject of numerous AI investigations [14–20]. AI landmark identification showed supremacy over traditional cephalometric landmark identification in terms of efficiency and repeatability [17–21]. 

Recently, AI has become the most studied digital technology in Orthodontics, as its integration into orthodontics is leading to a paradigm shift in the field. As AI continues to advance, it is expected to have a bigger impact on the future of orthodontics. Hence, it is imperative for orthodontists and dental students to cope with the latest advancements and to develop a solid understanding of the digital technologies [22]. Although AI-assisted lateral cephalometric analysis saves a lot of time prior to treatment, there are limitations to implementing this technology in every dental office, especially in low-income countries and the developing world. These limitations are closely related to the economic conflicts that often stem from limited resources. Such limited resources can lead to the inability to integrate advanced digital and AI systems in the daily dental practice [23]. To address these challenges, it is essential to adopt cost-effective solutions that make the most of the available resources. For that reason, it becomes substantial to evaluate the precision of these cost-effective freely available solutions and to compare their performance with the commercially available ones.

Multiple AI-based software for automatic cephalometric analysis has emerged recently, some of which are commercial and desktop-based while others are freely available and cloud-based [24, 25]. Despite the plethora of such software, there is a critical lack of direct comparisons in the literature between the free and the commercial software. To the best of our knowledge no studies have compared a freely available web-based platform for AI cephalometric tracing with both commercially available desktop-based software and gold standard manual cephalometric tracing. Therefore, the aim of the current study was to assess the performance of AI-assisted cephalometric analysis using a freely available web-based platform (WebCeph) by comparing it with the manual tracing (Gold standard) using OnyxCeph software (Image Instruments, Chemnitz, Germany). Moreover, it was compared with the commercially available desktop-based software (Carestream). The study’s null hypothesis was that there would be no significant difference in the agreement between the three methods of cephalometric tracing.

## Materials and methods

### Study design

The present study was a comparative in silico study and was conducted on 57 lateral cephalometric radiographs collected from Oral and Maxillofacial Radiology department, Faculty of Dentistry, Alexandria University. The study was approved by the Scientific Research Ethics Committee, Faculty of Dentistry, Alexandria University (IORG: 0008839), Ethics committee (No: 0933-06/2024). Cephalometric tracing was performed on the radiographs using 3 methods, one was considered as the control group and two were set as test groups. Digital manual tracing by OnyxCeph software was considered as the control group [26], freely available web-based AI cephalometric tracing platforms by WebCeph was set as test group 1 and commercial desktop-based AI software by Carestream was set as test group 2. The measurements of each method were then compared.

Radiographs of individuals with dental and skeletal Class I, II, or III malocclusion were included in the study, while radiographs of patients with any dentofacial disorder or congenital abnormality or individuals with mixed dentition were excluded from the study. Cephalometric radiographs with low quality and motion artifacts were also excluded, there was no restriction regarding patients’ ethnicity or gender when selecting the radiographs.

#### Sample size calculation

Sample size was estimated based on assuming confidence level = 95% and study power = 80%. According to Mahto et al. [27], the Intra Class Correlation Coefficient (ICC) value of cephalometric measurements for SNA between manual tracing and AI software was 0.879, with an ICC value of 0.75 was considered as the minimum value for good agreement [28]. The minimum sample size was calculated to be 51 digital lateral cephalometric images, this was increased to 57 digital images to make up for any processing errors. Sample size was based on Rosner’s method [29] calculated by Wnarifin Online Calculator for Intraclass Correlation Coefficient (ICC) - Hypothesis Testing [30]. 

### Data collection

Fifty seven pretreatment lateral cephalograms were obtained from the database of Oral and Maxillofacial Radiology department, Faculty of Dentistry, Alexandria University. The patients’ consent was obtained before the radiographic exposure and the patients granted permission for the use of the de-identified radiographs for research purposes. All the radiographs were taken with the patient standing upright while keeping the teeth in centric occlusion, the Frankfort plane parallel to the floor, and the lips in resting position. All the radiographs were acquired by Carestream CS 8100 X-ray machine (Carestream Dental LLC, Atlanta, GA, USA).

### Cephalometric analysis

#### Digital manual cephalometric analysis method (control group)

**Fig. 1:**
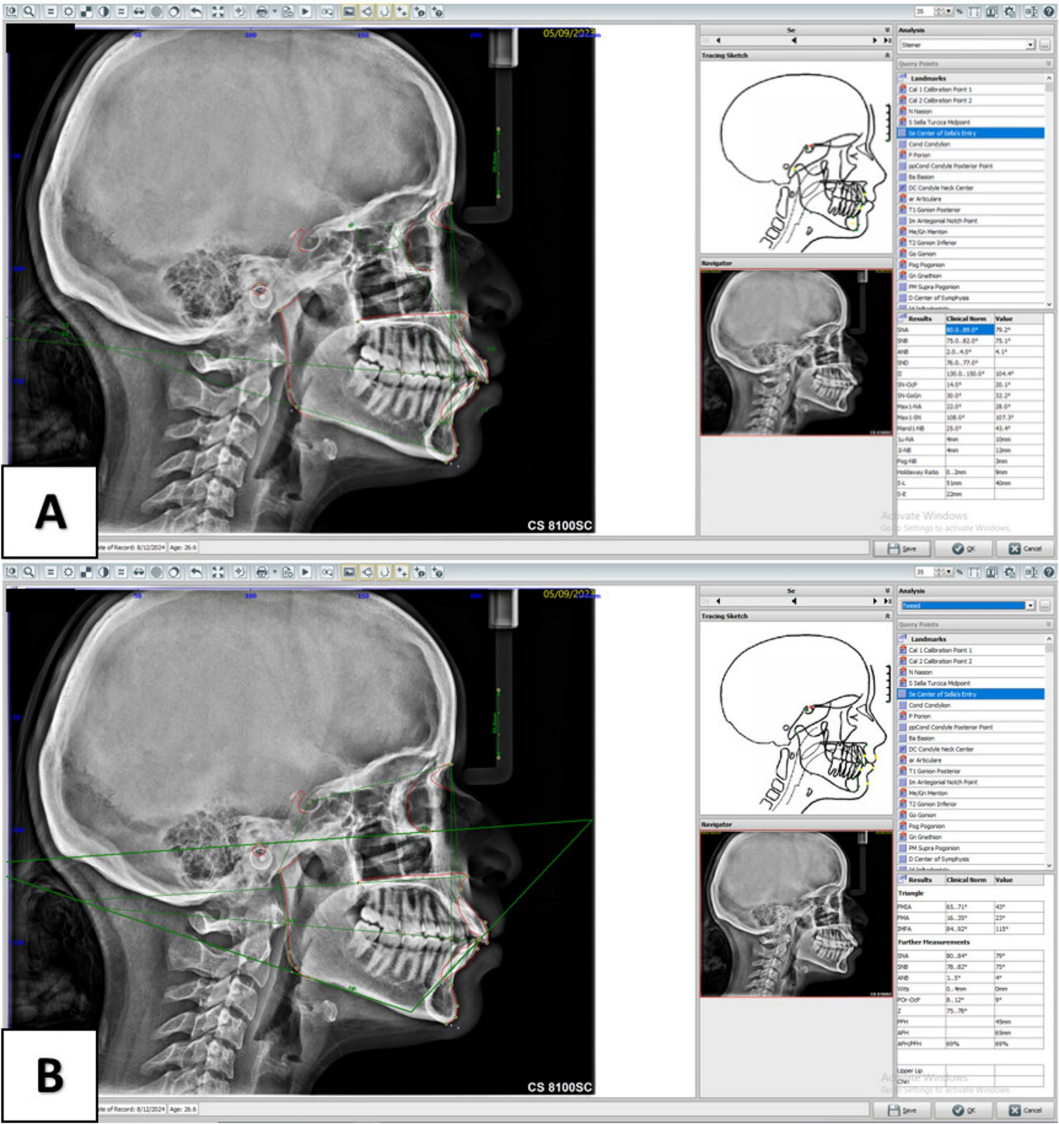
Steiner (**A**) and Tweed (**B**) analyses performed using OnyxCeph software showing landmark placement and measurement outputs

For this technique, all the JPG images of the radiographs were transferred to the OnyxCeph^3^™ (Image Instruments, Chemnitz, Germany) dental analysis software for cephalometric analysis. Digital measurements were evaluated using a Dell P2217Hb monitor. Consistency in landmark identification was ensured by clearly defining the selected anatomical landmarks before the study commencement [31]. The identified landmarks [Supplementary file 1] were marked using an indicator on the mouse control with any bilateral structure being averaged to make only one landmark. A ruler on the cephalostat was calibrated in the program before marking the anatomical points. Measurements were calculated automatically by the program after marking all the landmarks (Fig. 1).

All tracings were done by only one radiologist. No more than 6 radiographs were traced per day to prevent examiner fatigue. A total of 18 cephalometric measurements with 5 linear, 12 angular measurements and 1 ratio were evaluated. In these measurements, 11 parameters were skeletal and 7 parameters were dental. (Table 1) [31]. The abbreviations used by each software to denote the measured cephalometric readings are presented in Supplementary file 2.

**Table 1:** Measurements used in this study

Measurement	Definition
Steiner analysis	
SNA°	The angle between the SN plane and the NA line
SNB°	The angle between the SN plane and the NB line
ANB°	The angle between the NA and NB lines
II°	Interincisal angle measured as the angle between the most prominent maxillary and mandibular incisors
SN-OcP°	The angle between SN plane and the occlusal plane
SN-GoGn°	Mandibular plane angle measured as the angle between the mandibular plane (Gonion-Gnathion) and SN plane
Max1-NA°	The angle formed by the intersection of the NA line with the maxillary incisor long axis
Mand1-NB°	The angle formed by the intersection of the NB line with the mandibular incisor long axis
1u-NA (mm)	The linear distance from the incisal edge of the maxillary central incisor to NA line
1 L-NB (mm)	The linear distance from the incisal edge of the mandibular central incisor to NB line
Pog-NB (mm)	The linear distance from the pognion point to the NB line
Tweed analysis	
FMIA°	The angle formed between the Frankfort horizontal plane and long axis of mandibular incisor
FMA°	Mandibular plane angle measured as the angle between the Frankfort horizontal plane and the mandibular plane
IMPA°	The angle formed between the mandibular plane and the long axis of the mandibular incisor
POr-OcP°	Cant of the occlusal plane measured as the angle between the Frankfort horizontal plane and the occlusal plane
PFH (mm)	Posterior facial height measured as the linear distance from the Articulare point to the Gonion point
AFH (mm)	Anterior facial height measured as the linear distance between Menton and a line connecting ANS and PNS
AFH/PFH	Facial height index calculated as the ratio between the PFH to the AFH

#### Web-based AI cephalometric analysis method (test group 1)

**Fig. 2:**
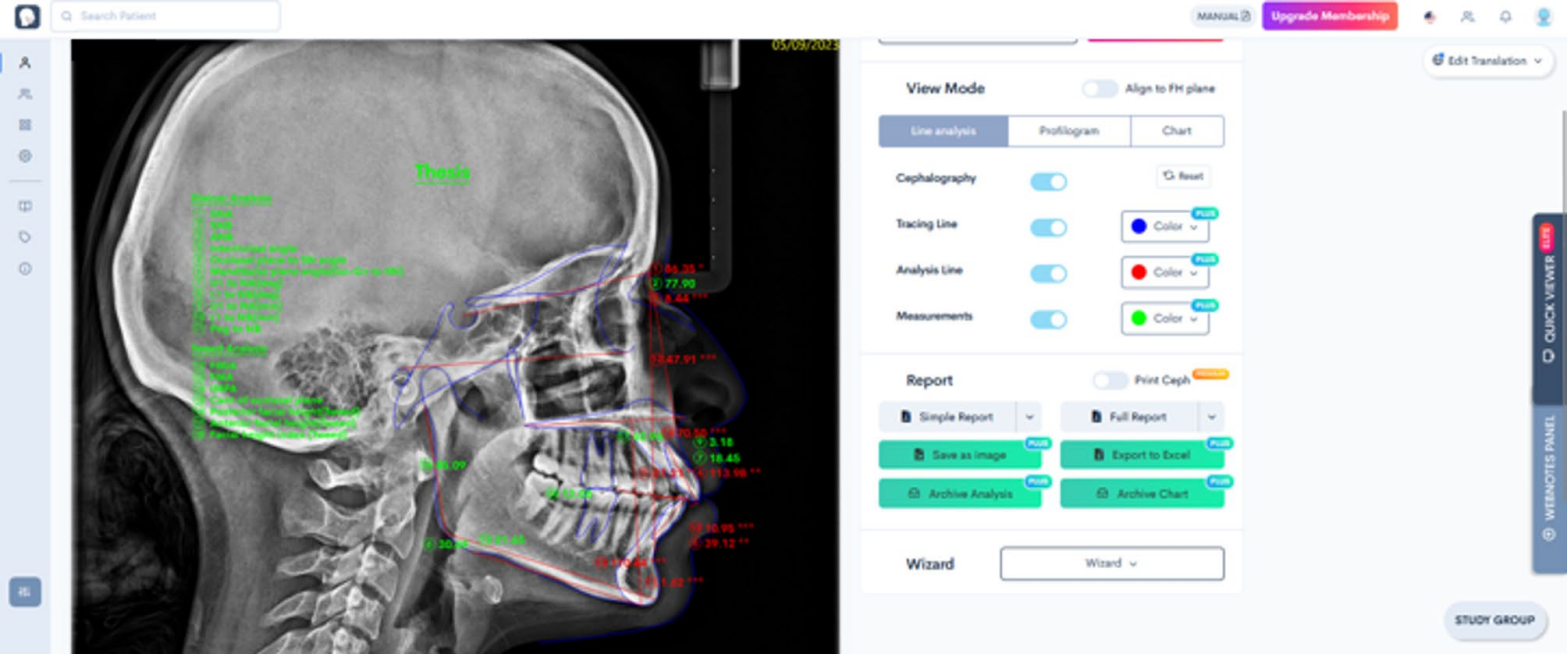
Steiner and Tweed analyses in Webceph platform showing automatic landmark placement and measurement outputs

For this technique the same radiographs were uploaded to the WebCeph™ (Artificial Intelligence Orthodontic & Orthognathic Cloud Platform, South Korea), free online cephalometric analysis service. A custom analysis was done on this platform to produce all the measurements simultaneously (Fig. 2).

For this method radiographs were only manually calibrated using a ruler on the cephalostat, then all the cephalometric landmarks were identified and digitized and measurements produced automatically by AI without any human intervention.

#### Desktop-based AI cephalometric analysis method (test group 2)

**Fig. 3:**
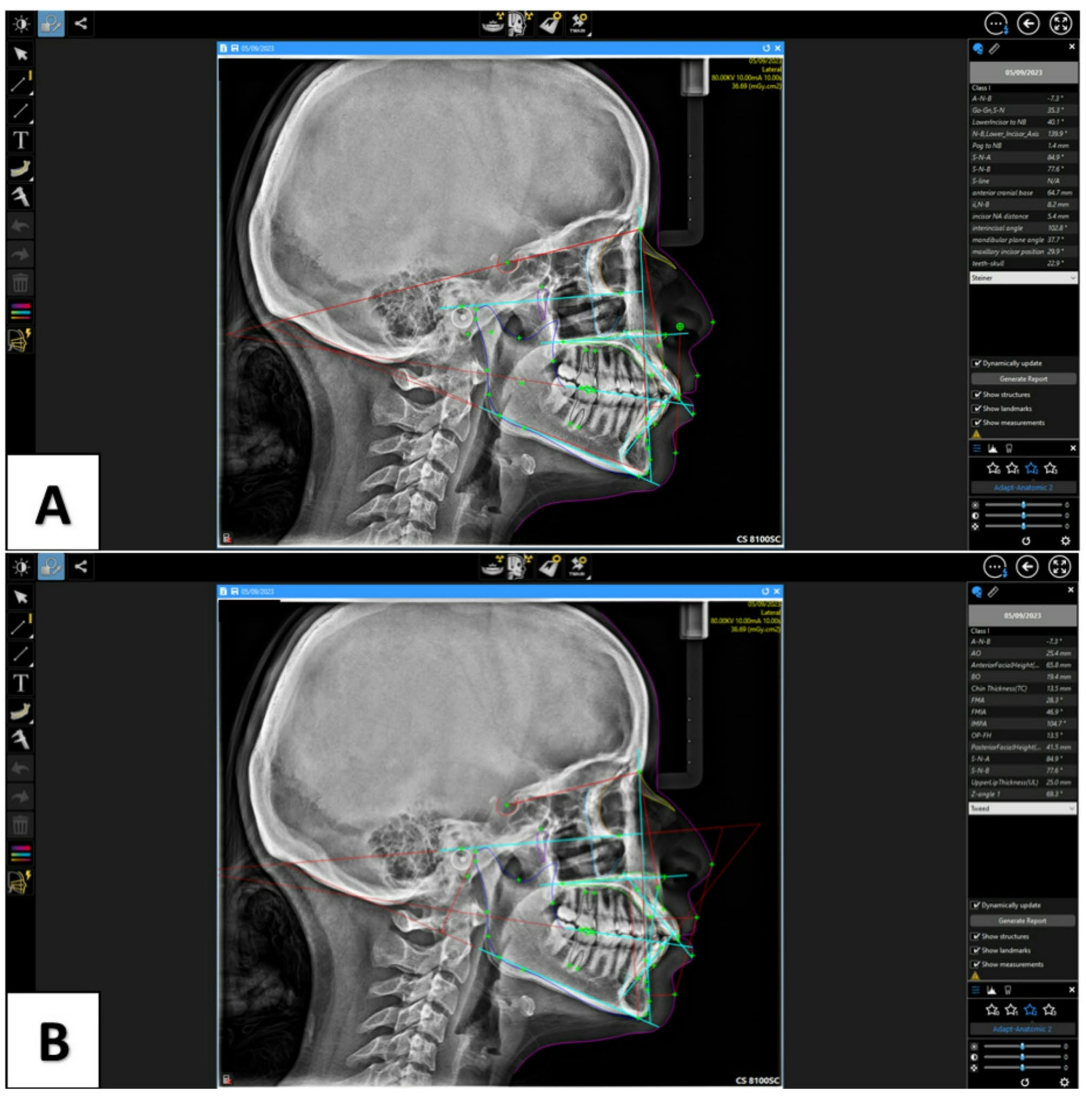
Steiner (**A**) and Tweed (**B**) analyses performed using Carestream software showing landmark placement and measurement outputs

For this technique Carestream imaging software V8 (Carestream Dental LLC, Atlanta, GA, USA) was used. All radiographs were only manually calibrated using a ruler on the cephalostat. All the cephalometric landmarks were identified and digitized and measurements produced automatically by AI without any human intervention (Fig. 3).

For the two AI groups, the cephalometric analysis process was fully automated as the software placed all landmarks without any manual correction or intervention from the operator.

### Reliability assessment

Before the study commencement, the researcher who performed the manual cephalometric tracing (OE) was calibrated by repeatedly performing the cephalometric analysis to ensure correct placement of the landmarks and the landmark positions were validated by an experienced orthodontist (YY).

During the study, 12 images were randomly chosen and retraced by the same researcher and by a different researcher, with a 2-week interval between assessments to assess intra- and inter-examiner reliability, respectively.

### Blinding

The researcher manually placed the cephalometric landmarks only in OnyxCeph software, therefore, blinding of the researcher during landmark identification was not possible. Moreover, because cephalometric measurements were automatically generated using the three software, there was no chance of human bias influencing the results, thus negating the need for blinding during this step. Nevertheless, while operator blinding was not possible, the order of analysis in the different software was randomized to prevent systematic bias. In addition, the statistician was blinded during data assessment.

### Statistical analysis

Normality of all parameters were checked using Shapiro Wilk test and Q-Q plots. Normal distribution was approved for all parameters except ANB, 1u-NA, 1 L-NB and Pog-NB. All data were presented using mean, standard deviation (SD) in addition to median and inter quartile range (IQR). Repeated measures ANOVA and Friedman test followed by pairwise comparisons with Bonferroni correction to adjust for type I error was used for comparison between groups. Mean differences and 95% Confidence intervals were calculated. Intra and inter examiner reliability were examined using Intra Class Correlation Coefficient (ICC). In addition, ICC was used to determine the agreement between the measurements obtained using the three methods of cephalometric analysis. All tests were two tailed and the significance level was set at p value < 0.05. Data was analyzed using IBM SPSS, for Windows, version 23, Armonk, NY, USA.

## Results

The ICC values showed intra-examiner reliability ranging between (0.768–0.993) for all measurements indicative of good to excellent intra-observer reliability [28]. The ICC of the mean measurement values between the two researchers ranged between (0.895–0.996) also indicative of good to excellent inter-observer reliability with the exception of 1u-NA which was (0.741) indicating moderate inter-observer reliability for this measurement (Table 2) [28]. 

**Table 2:** Inter-examiner reliability and intra-examiner reliability of all parameters

	Intra-examiner			Inter-examiner		
	ICC	95% CI	P value	ICC	95% CI	P value
SNA	0.979	0.928, 0.994	< 0.001*	0.944	0.891, 0.984	< 0.001*
SNB	0.987	0.954, 0.996	< 0.001*	0.929	0.774, 0.979	< 0.001*
ANB	0.982	0.940, 0.995	< 0.001*	0.972	0.905, 0.992	< 0.001*
II	0.988	0.961, 0.997	< 0.001*	0.989	0.961, 0.997	< 0.001*
SN-OcP	0.961	0.871, 0.989	< 0.001*	0.975	0.916, 0.993	< 0.001*
SN-GoGn	0.983	0.944, 0.995	< 0.001*	0.963	0.878,0.989	< 0.001*
Max1-NA	0.973	0.910, 0.992	< 0.001*	0.976	0.918, 0.993	< 0.001*
Mand1-NB	0.991	0.970, 0.998	< 0.001*	0.990	0.965, 0.997	< 0.001*
1u-NA	0.768	0.375, 0.927	0.001*	0.741	0.319, 0.918	< 0.001*
1 L-NB	0.919	0.743, 0.976	< 0.001*	0.962	0.874, 0.989	< 0.001*
Pog-NB	0.857	0.578, 0.957	< 0.001*	0.895	0.678, 0.969	< 0.001*
FMIA	0.993	0.977, 0.998	< 0.001*	0.991	0.968, 0.997	< 0.001*
FMA	0.944	0.819, 0.984	< 0.001*	0.944	0.817, 0.983	< 0.001*
IMPA	0.992	0.973, 0.998	< 0.001*	0.990	0.966, 0.997	< 0.001*
POr-OcP	0.908	0.712, 0.972	< 0.001*	0.926	0.764, 0.978	< 0.001*
PFH	0.960	0.868, 0.988	< 0.001*	0.962	0.873, 0.989	< 0.001*
AFH	0.988	0.957, 0.996	< 0.001*	0.996	0.986, 0.999	< 0.001*
AFH/PFH	0.978	0.927, 0.994	< 0.001*	0.975	0.915, 0.993	< 0.001*

*ICC* Intra Class Coefficient, *CI* Confidence Interval

*Statistically significant difference at p value < 0.05

Comparison of the mean cephalometric measurements revealed significant differences between the three methods of cephalometric tracing. The results for Steiner and Tweed analysis are shown in Tables 3 and 4, respectively. Pairwise comparisons of Steiner measurements among the three software programs are shown in Table 5. The results showed that the magnitude of the mean differences between both AI software versus manual tracing was generally below the clinical threshold of 2 mm/degrees, except for Max1-NA and Mand1-NB. Pairwise comparisons of Tweed measurements among the three software programs are shown in Table 6. The results demonstrated significant differences in 5 parameters between Carestream software and the manual gold standard that considerably exceeded the 2 mm/degrees clinical threshold (FMA, IMPA, Por-OcP, PFH, AFH/PFH), whereas the differences between WebCeph and the manual gold standard were not significant. The ICC values of the cephalometric measurements obtained using the three methods are reported in supplementary file 3. For the values of WebCeph versus manual tracing, all the measurements showed ICC values above 0.8, indicating good to excellent agreement [28], except 1u-NA which showed an ICC value of 0.529. On the other hand, the ICC values of Carestream versus manual tracing ranged between 0.386 and 0.833, indicating poor to good agreement [28], with only the AFH showing an ICC value of 0.970.

**Table 3:** Comparison of Steiner measurements between Onyxceph, Carestream and Webceph

	**Control group**	**Test groups**		
	Onyxceph(n=57)	Carestream(n=57)	Webceph(n=57)	P value
	Mean ±SD	Mean ±SD	Mean ±SD	
SNA°	82.26 ±4.90	83.07±5.02	82.85±3.49	0.467
SNB°	77.63 ± 4.36	78.09 ± 7.04	77.37 ± 3.52	0.534
ANB°^ⱡ^	4.64 ± 3.66	4.46 ± 3.34	5.47 ± 3.29	< 0.001*
II°	126.38 ± 13.02	126.47 ± 20.35	129.89 ± 11.47	0.171
SN-OcP°	19.15 ± 6.23	17.09 ± 7.84	19.39 ± 5.87	0.035*
SN-GoGn°	34.86 ± 5.98	35.12 ± 7.02	33.96 ± 6.81	0.214
Max1-NA°	20.34 ± 8.68	21.36 ± 7.84	17.95 ± 7.75	0.002*
Mand1-NB°	28.69 ± 8.04	25.62 ± 8.21	26.68 ± 7.27	0.006*
1u-NAmm^ⱡ^	4.33 ± 2.93	3.51 ± 2.22	2.68 ± 2.07	< 0.001*
1 L-NBmm^ⱡ^	5.88 ± 2.54	5.30 ± 2.44	6.09 ± 2.83	0.063
Pog-NBmm^ⱡ^	1.04 ± 1.45	1.22 ± 0.86	1.05 ± 1.55	0.520

ⱡFriedman Test, all other p values are obtained from Repeated Measures ANOVA

*Statistically significant difference at p value < 0.05

**Table 4:** Comparison of Tweed measurements between Onyxceph, Carestream and Webceph

	Control group	Test groups		
	Onyxceph (n = 57)	Carestream (n = 57)	Webceph (n = 57)	P value^ⱡ^
	Mean ± SD	Mean ± SD	Mean ± SD	
FMIA°	57.09 ± 8.94	57.05 ± 8.64	59.45 ± 8.21	0.049*
FMA°	28.30 ± 6.68	33.29 ± 5.43	26.52 ± 6.27	< 0.001*
IMPA°	94.63 ± 9.48	89.61 ± 8.66	94.04 ± 9.38	< 0.001*
POr-OcP°	10.96 ± 5.20	13.13 ± 5.71	10.65 ± 4.30	0.001*
PFHmm	41.75 ± 5.71	38.35 ± 2.56	40.89 ± 5.12	< 0.001*
AFHmm	61.32 ± 6.92	61.83 ± 5.78	61.47 ± 6.88	0.120
AFH/PFH	68.60 ± 9.16	62.32 ± 4.62	66.83 ± 7.44	< 0.001*

ⱡRepeated Measures ANOVA

*Statistically significant difference at p value < 0.05

**Table 5:** Pairwise comparisons and mean differences of Steiner measurements between Onyxceph, Carestream and Webceph

Parameters	Groups	Compared to	Mean diff	95% CI	*p* value
SNA°	Onyxceph	Carestream	−0.81	−2.84, 1.22	-
		Webceph	−0.58	−1.71, 0.54	-
	Carestream	Webceph	0.23	−1.47, 1.92	-
SNB°	Onyxceph	Carestream	−0.47	−2.83, 1.90	-
		Webceph	0.25	−0.49, 0.99	-
	Carestream	Webceph	0.72	−1.46, 2.89	-
ANB°	Onyxceph	Carestream	0.18	−0.79, 1.14	1.00
		Webceph	−0.84	−1.54, −0.14	0.001*
	Carestream	Webceph	−1.01	−1.72, −0.30	< 0.001*
II°	Onyxceph	Carestream	−0.09	−6.31, 6.12	-
		Webceph	−3.51	−5.67, −1.35	-
	Carestream	Webceph	−3.42	−9.20, 2.36	-
SN-OcP°	Onyxceph	Carestream	2.05	−0.60, 4.71	0.183
		Webceph	−0.25	−1.21, 0.72	1.00
	Carestream	Webceph	−2.30	−4.68, 0.08	0.061
SN-GoGn°	Onyxceph	Carestream	0.25	−1.81, 2.32	-
		Webceph	1.16	0.25, 2.07	-
	Carestream	Webceph	0.91	−0.94, 2.75	-
Max1-NA°	Onyxceph	Carestream	−1.03	−3.58, 1.53	0.980
		Webceph	2.38	0.31, 4.46	0.019*
	Carestream	Webceph	3.41	1.05, 5.77	0.002*
Mand1-NB°	Onyxceph	Carestream	3.07	0.60, 5.54	0.010*
		Webceph	2.01	0.88, 3.14	< 0.001*
	Carestream	Webceph	−1.06	−3.45, 1.33	0.839
1u-NAmm	Onyxceph	Carestream	0.83	−0.12, 1.77	0.569
		Webceph	1.65	0.72, 2.59	< 0.001*
	Carestream	Webceph	0.83	0.19, 1.47	0.030*
1 L-NBmm	Onyxceph	Carestream	0.57	−0.04, 1.19	-
		Webceph	−0.22	−0.62, 0.19	-
	Carestream	Webceph	−0.79	−1.45, −0.13	-
Pog-NBmm	Onyxceph	Carestream	−0.19	−0.50, 0.13	-
		Webceph	−0.02	−0.29, 0.25	-
	Carestream	Webceph	0.17	−0.16, 0.50	-

*CI* Confidence Interval

*Statistically significant difference at p value < 0.05

**Table 6:** Pairwise comparisons and mean differences of tweed measurements between Onyxceph, Carestream and Webceph

Parameters	Groups	Compared to	Mean diff	95% CI	*p* value
FMIA°	Onyxceph	Carestream	0.04	−3.06, 3.13	1.00
		Webceph	−2.36	−3.64, −1.08	< 0.001*
	Carestream	Webceph	−2.39	−5.12, 0.33	0.104
FMA°	Onyxceph	Carestream	−4.99	−6.72, −3.27	< 0.001*
		Webceph	1.78	0.58, 2.98	0.002*
	Carestream	Webceph	6.77	5.23, 8.31	< 0.001*
IMPA°	Onyxceph	Carestream	5.03	2.27, 7.79	< 0.001*
		Webceph	0.60	−0.78, 1.97	0.865
	Carestream	Webceph	−4.43	−7.29, −1.58	0.001*
POr-OcP°	Onyxceph	Carestream	−2.17	−4.14, −0.19	0.027*
		Webceph	0.32	−0.88, 1.52	1.00
	Carestream	Webceph	2.48	1.00, 3.96	< 0.001*
PFHmm	Onyxceph	Carestream	3.40	1.99, 4.81	< 0.001*
		Webceph	0.87	−0.21, 1.95	0.155
	Carestream	Webceph	−2.55	−3.83, −1.24	< 0.001*
AFHmm	Onyxceph	Carestream	−0.51	−1.22, 0.20	-
		Webceph	−0.15	−0.60, 0.30	-
	Carestream	Webceph	0.36	−0.28, 1.00	-
AFH/PFH	Onyxceph	Carestream	6.28	3.84, 8.71	< 0.001*
		Webceph	1.77	−0.14, 3.67	0.078
	Carestream	Webceph	−4.51	−6.52, −2.50	< 0.001*3

*Statistically significant difference at p value < 0.05

## Discussion

Cephalometric analysis is an integral part of orthodontic treatment planning. Conventionally, manual cephalometric analysis is performed using acetate paper, ruler and protractor. However, it has limited reproducibility upon repetition and consumes too much time [3]. Currently, the use of AI for automatic tracing of cephalometric radiographs is showing great promise to overcome the limitations of conventional manual tracing [27]. Although a previous study showed that AI-automated cephalometric analysis was less accurate and slower compared to manual tracing performed by skilled clinicians, the ongoing technological advances continue to improve the performance of AI [32]. Recent research has demonstrated that automatic cephalometric analysis is superior in terms of repeatability, and it saves time as manual tracing takes almost 80 times as long as the AI software takes to trace a cephalogram [1]. Consequently, a number of fully automated AI-driven cephalometric analysis software and platforms have been developed [33, 34]. 

However, economic issues, especially in low-income countries and governmental institutions, hinder the utilization of commercially available AI-assisted software and their periodic license renewals present an additional burden that cannot be afforded most of the time. Little data is available in the literature about the precision of freely available platforms for automatic cephalometric analysis and their applicability as alternatives to the commercial software. This study aimed to assess the performance of AI-assisted cephalometric analysis using a freely available web-based platform in comparison to manual tracing, and to compare its performance with another commercially available desktop-based software. Significant differences were found between the three methods of tracing, hence, the null hypothesis was rejected. The free platform, WebCeph, performed comparably to manual tracing, and outperformed the commercial software, Carestream, in several key parameters.

The choice to compare measurements, not landmark identification, was made because they are the end products of the cephalometric analysis process, which provides data to aid in treatment planning [35]. OnyxCeph has been shown in a previous study [26] to be an efficient software to perform cephalometric analysis, therefore it was used as the gold standard for manual cephalometric tracing in the current study. The aforementioned study showed that minimal statistical differences were detected between the measurements generated by conventional manual tracing and those generated by digital tracing using OnyxCeph program.

The comparison between the three software in the current study was limited to Steiner and Tweed analysis. The choice of the cephalometric parameters included in the study was based on their availability in the three software. In addition, to ensure consistency between the three methods, the description of the measured cephalometric parameters in each software was checked for conformity prior to the study commencement. When interpreting the results, a difference of < 2 mm/degrees was considered clinically acceptable based on previous published research [26, 36–38]. 

In the current study, good to excellent intra-examiner and inter-examiner reliability was reported for all the measured parameters, except 1u-NA, which showed moderate inter-examiner reliability. Such disparity possibly pertains to the difficulty in the precise localization of the incisal edge landmark in cases where the maxillary central incisor is rotated.

The agreement of the measured parameters between the free web-based automatic platform (WebCeph) and the manual gold standard was generally good to excellent. Similarly, Mahto et al. [38] reported good to excellent agreement between WebCeph platform and conventional manual tracing. In the current study, WebCeph differed significantly from both OnyxCeph (the gold standard) and Carestream only regarding ANB, Max1-NA, Mand1-NB, 1u-NA and FMIA. The differences regarding Max1-NA and FMIA may be attributed to the difficulty of tracing the exact outline of the incisors due to the overlapping of the images of the anterior teeth in the cephalometric radiograph. Another reason may be the difficulty faced by AI to accurately locate the Or and Po points of the Frankfort horizontal plane since these points lie on indistinct areas that are not as well-defined as a suture or a sharp bony angle. The current study results are similar to the results of previous research [39, 40] which applied the same inclusion and exclusion criteria and included Onyxceph software as the gold standard. On the other hand, the results of Mercier et al. [36] and El-Dawlatly et al. [39] studies found Webceph to be less accurate than the manual gold standard method. A possible reason for the improved accuracy of WebCeph in the current study is that the AI algorithm has been trained over time thus allowing it to identify landmarks more precisely and to generate more accurate results. The clinical significance of the variations detected between the cephalometric measurements acquired using manual versus AI landmark identification is an important aspect to consider [26, 36–38]. Despite the reported statistical significance regarding ANB, Mand1-NB and 1u-NA, the mean differences of the measurements between the two tracing methods was less than 2 mm/degrees, which is considered clinically non-significant.

As for the commercial desktop-based AI software, poor to good agreement was found between Carestream and the manual gold standard for most of the measured parameters. The results showed that there were significant differences between Carestream and both OnyxCeph (the gold standard) and Webceph in SN-OcP, Mand1-NB, POr-OcP, and IMPA. These variations may be explained by the different methods each software uses to identify the occlusal plane. Moreover, as mentioned earlier, the difficulty in identification of the Frankfort horizontal plane may have contributed to these differences. The overlapping of the incisors in the anterior region makes their identification by either the manual or the automated method challenging. Moreover FMA, PFH, and PFH to AFH ratio measurements in Carestream differd significantly from both OnyxCeph and WebCeph measurements. A possible reason may be the occurrence of an error in localization of Go point, which is a bilateral structure, thus affecting the mandibular plane inclination and the PFH. When the right and left sides do not overlap, the midpoint between them is arbitrarily selected, thus possibly introducing errors in the related measurements. Go point localization may also be impacted by obstruction by surrounding anatomical structures which increases the difficulty of its detection by AI. AI systems are trained to detect patterns. However, for areas that are obscured by the surrounding structures or the overlapping structures, AI may find it harder to learn a single consistent pattern resulting in higher rates of error. Conversely, the human evaluator can rely on their personal judgment and experience to accurately determine the location of cephalometric points [27, 41]. It was also found that when using AI, if a landmark was on a well-delineated border of the cephalometric structure or a structure with high contrast, then the mean error would be less than if it was in an area that was not well delineated or in a low contrast area of the cephalometric radiograph [42]. The results of the current study are partially in agreement with previous research [43] that found significant differences between automated tracing using Carestream software and manual tracing using Dolphin 3D Imaging software regarding FMA and IMPA. However, unlike the current research, the previous study generally reported good agreement between Carestream automated tracing and manual tracing [43]. The inconsistency between the study results may have arisen from the different study settings. The radiographs in the abovementioned study were obtained from a private clinic, whereas the radiographs in the current study were collected from the archives of a University hospital where more complex cases are commonly encountered [44]. In complex malocclusions, the overlapping structures and severe skeletal deformities may preclude the accurate detection of cephalometric landmarks using AI.

In addition to being free to use, another possible advantage of the web-based system evaluated in the current study is that it eliminates the need for the time-consuming software installation procedures, and provides cloud storage of the patients’ orthodontic records, hence conserving digital storage space on personal computers and enabling access to the records from any operating system or device [45]. Additionally, being cloud-based eliminates the financial burden of buying high-performance computers with specific hardware requirements that are needed to run complex desktop software [46]. On the other hand, for orthodontists in areas with slow internet connection, a cloud-based system may present a significant drawback. In such case, a desktop-based program, once installed, can be used offline, making it more practical for operators with limited internet access. Webceph platform also provides a semi-automatic cephalometric analysis option where the operator can edit the placement of landmarks after automatic digitization by AI and it was stated that this semi-automatic method is more efficient than the fully automatic one [36, 39]. Based on the results of the current study, the accuracy of the free AI cephalometric analysis software may be considered acceptable and surpasses the commercial software. Nevertheless human supervision is still necessary after AI- assisted cephalometric analysis [27]. Additionally the accuracy of cephalometric measurements might increase if the quality of the images is improved by modifying sharpness and contrast [33]. 

A primary limitation of the current study is that only one software was used to represent each cephalometric tracing method, consequently limiting the generalizability of the study results. Moreover, only two cephalometric analyses (Steiner and Tweed) were evaluated in the current study. The accuracy of the tested software in performing other types of cephlometric analyses and the performance of other similar software should be assessed in future studies. Another factor that limits the generalizability of the results is the exclusion of radiographs of patients in the mixed dentition stage and patients with craniofacial anomalies. Furthermore, the performance of the AI software highly depends on the quality of the radiographic images. In the current study, images with low quality and radiographs showing motion artifacts were excluded. Future research should investigate the performance of AI software using a more diverse sample representing the real-world clinical situtaion. An additional limitation of the current study is that a single operator performed the manual tracings. Despite calibrating the researcher prior to the study commencement, this may have introduced bias. Another recommendation for future research is to incorporate 3D cephalometrics as the current study only assessed 2D cephalometric imaging.

Apart from cephalometric analysis, AI is now being implemented in face analysis and enhancement, which has potential applications in orthodontic treatment planning. For example, a recent study has demonstrated that AI was able to enhance the facial features of patients, thus guiding the orthodontists in formulating personalized treatment plans that address the patients’ esthetic concerns [47]. Additionally, some of those AI-powered apps run on smartphones, making them transportable and easily accessible, hence we recommend that these applications to be investigated in future studies.

## Conclusions

Within the limitations of the current study, it was concluded that AI-assisted cephalometric analysis using the freely available web-based platform, WebCeph was more precise than AI-assisted cephalometric analysis using the commercially available desktop-based software, Carestream. The accessibility and precision of WebCeph could allow orthodontists to efficiently perform cephalometric analysis particularly in resource-limited settings where commercial software is prohibitively expensive. Human supervision over AI-assisted cephalometric tracing is advised.

## Supplementary Information

Supplementary Material 1: Supplementary file 1. Landmarks that were identified in Onyxceph software

Supplementary Material 2: Supplementary file 2. Names of the measured parameters in each software

Supplementary Material 3: Supplemental Table 3. ICC values of cephalometric measurements between the three methods of cephalometric analysis

## Data Availability

The datasets used or analyzed during the current study are available from the corresponding author on reasonable request.

## Abbreviations

AI: Artificial intelligence
ICC: Intra Class Correlation Coefficient
SD: Standard deviation
