# Supplementary material for: Evaluating a cost-effective, web-based AI platform for lateral cephalometric analysis: a comparative in-silico study
Source: BMC Oral Health. 2025 Dec 18;26:7. doi: 10.1186/s12903-025-07108-6 (PMC12766963; doi:10.1186/s12903-025-07108-6)
Supplement: Supplementary file 1 — Supplementary Material 1: Supplementary file 1. Landmarks that were identified in Onyxceph software [file 12903_2025_7108_MOESM1_ESM.docx]

Supplementry table 1: Landmarks that was identified in Onyxceph software

| Nasion (N) |
| --- |
| Sella (S) |
| Porion (P) |
| Articular (ar) |
| Gonion posterior (T1) |
| Gonion inferior (T2) |
| Me/Gn Menton |
| Gonion (Go) |
| Pogonion (Pog) |
| Gnathion (Gn) |
| A-Point (A) |
| B-Point (B) |
| Anterior nasal spine (ANS) |
| Posterior nasal spine (PNS) |
| Orbitale (Or) |
| Upper incisor apex (Ap 1u) |
| Upper incisor labial outline (Ls 1u) |
| Upper incisor crown tip (Is 1u) |
| Lower incisor crown tip (Is 1l) |
| Lower incisor labial outline (Li 1l) |
| Lower incisor apex (Ap 1l) |
| Occlusal plane posterior point (ppOcP) |
| Occlusal plane anterior point (apOcP) |
