# Supplementary material for: Evaluating a cost-effective, web-based AI platform for lateral cephalometric analysis: a comparative in-silico study
Source: BMC Oral Health. 2025 Dec 18;26:7. doi: 10.1186/s12903-025-07108-6 (PMC12766963; doi:10.1186/s12903-025-07108-6)
Supplement: Supplementary file 2 — Supplementary Material 2: Supplementary file 2. Names of the measured parameters in each software [file 12903_2025_7108_MOESM2_ESM.docx]

Supplementry table 2: Measurements' names in each software

| **Onyxceph** | **Carestream** | **Webceph** |  |
| --- | --- | --- | --- |
| SNA | S-N-A | SNA | The angle between the SN plane and the NA line |
| SNB | S-N-B | SNB | The angle between the SN plane and the NB line |
| ANB | A-N-B | ANB | The angle between the NA and NB lines |
| II | interincisal angle | Interincisal angle | The angle between the most prominent maxillary and mandibular incisors |
| SN-OcP | teeth-skull | Occlusal plane to SN angle | The angle between SN plane and the occlusal plane |
| SN-GoGn | Go-Gn,S-N | Mandibular plane angle (Go-Gn to SN) | The angle between the mandibular plane (Gonion-Gnathion) and SN plane |
| Max1-NA | maxillary incisor position | U1 to NA (deg) | The angle formed by the intersection of the NA line with the maxillary incisor long axis |
| Mand1-NB | LowerIncisor to NB | L1 to NB (deg) | The angle formed by the intersection of the NB line with the mandibular incisor long axis |
| 1u-NA | incisor NA distance | U1 to NA (mm) | The distance from the incisal edge of the maxillary central incisor to NA line |
| 1l-NB | ii,N-B | L1 to NB (mm) | The distance from the incisal edge of the mandibular central incisor to NB line |
| Pog-NB | Pog to NB | Pog to NB | The distance from the pognion point to the NB line |
| FMIA | FMIA | FMIA | The angle formed between the Frankfort horizontal plane and long axis of mandibular incisor |
| FMA | FMA | FMA | The angle between the Frankfort horizontal plane and the mandibular plane |
| IMPA | IMPA | IMPA | The angle formed between the mandibular plane and the long axis of the mandibular incisor |
| POr-OcP | OP-FH | Cant of occlusal plane | The angle between the Frankfort horizontal plane and the occlusal plane |
| PFH | PosteriorFacialHeight | Posterior facial height (Tweed) | The distance from the Articulare point to the Gonion point |
| AFH | AnteriorFacialHeight | Anterior facial height (Tweed) | The distance between Menton and a line connecting ANS and PNS |
| AFH/PFH | "Calculated Ratio" | Facial height index (Tweed) | The ratio between the PFH to the AFH |
