# Supplementary material for: Evaluating a cost-effective, web-based AI platform for lateral cephalometric analysis: a comparative in-silico study
Source: BMC Oral Health. 2025 Dec 18;26:7. doi: 10.1186/s12903-025-07108-6 (PMC12766963; doi:10.1186/s12903-025-07108-6)
Supplement: Supplementary file 3 — Supplementary Material 3: Supplemental Table 3. ICC values of cephalometric measurements between the three methods of cephalometric analysis [file 12903_2025_7108_MOESM3_ESM.docx]

Supplemental table 3: ICC values of cephalometric measurements between the three methods of cephalometric analysis.

|  |  | Onyxceph – WebCeph | Onyxceph – Carestreem | Carestream – WebCeph |
| --- | --- | --- | --- | --- |
| Steiner  measurements | SNA | 0.804 | 0.435 | 0.435 |
|  | SNB | 0.912 | 0.386 | 0.447 |
|  | ANB | 0.896 | 0.783 | 0.880 |
|  | II | 0.922 | 0.552 | 0.599 |
|  | SN-OcP | 0.937 | 0.512 | 0.619 |
|  | SN-GoGn | 0.919 | 0.694 | 0.758 |
|  | Max1-NA | 0.826 | 0.713 | 0.728 |
|  | Mand1-NB | 0.946 | 0.725 | 0.714 |
|  | 1u-NA | 0.529 | 0.550 | 0.738 |
|  | 1l-NB | 0.945 | 0.833 | 0.828 |
|  | Pog-NB | 0.919 | 0.807 | 0.808 |
| Tweed measurements | FMIA | 0.945 | 0.593 | 0.676 |
|  | FMA | 0.911 | 0.763 | 0.807 |
|  | IMPA | 0.948 | 0.724 | 0.694 |
|  | POr-OcP | 0.827 | 0.557 | 0.749 |
|  | PFH | 0.898 | 0.688 | 0.685 |
|  | AFH | 0.990 | 0.970 | 0.976 |
|  | AFH/PFH | 0.862 | 0.643 | 0.673 |
